# Targeting Immuno-metabolism and Antiviral Immune Responses in Chronic Hepatitis B

**DOI:** 10.1007/s12072-023-10546-5

**Published:** 2023-06-27

**Authors:** Suzanne Faure-Dupuy, Thomas F Baumert

**Affiliations:** 1 Institut Cochin, Université de Paris, Inserm, U1016, CNRS, UMR 8104, Paris, F-75014, France; 2 Université de Strasbourg, Inserm, Institut de Recherche sur les Maladies Virales et Hepatiques UMR_S1110, Strasbourg; 3 Service d’hépato-gastroentérologie, Hôpitaux Universitaires de Strasbourg, Strasbourg; 4 Institut hospitalo-universitaire (IHU), Université de Strasbourg, Strasbourg; 5 Institut Universitaire de France (IUF), Paris

Chronic hepatitis B (CHB), caused by the hepatitis B virus (HBV), is a worldwide burden affecting up to 300 million patients and associated with the development of cirrhosis and hepatocellular carcinoma (HCC) [1]. The approved treatments for CHB patients comprise on nucleos(t)ide analogues (NA) and pegylated interferon alpha (Peg-IFNα) [1]. Whereas NA efficiently suppress replication, they rarely lead to viral eradication and do not fully prevent virus-induced HCC [1]. The use of Peg-IFN*α* is limited by adverse effects. Therefore, novel treatment approaches for viral cure are needed. Combination therapies targeting the viral life cycle (e. g. RNAi, capsid inhibitors) and activation of the antiviral immune response (e.g. pattern recognition receptors agonists, restoration of T cell responses) are currently under clinical investigation [1].

CHB has been associated with T-cell exhaustion, leading to weak and delayed T-cell responses, which are not sufficient to eliminate the virus [2,3]. Therefore, restoring T-cell function has emerged as a therapeutic candidate strategy [2,3]. In the last decade, important advances have been made to understand the interplay between immune responses and metabolism. Intrinsic cell metabolism has been shown to be a key regulator of immune responses, such as controlling the extent of the responses, the capacity to respond, and the type of response (i.e., pro- or anti-inflammatory) [4]. Indeed, the immuno-metabolism field has unravelled numerous previously unknown immune regulatory pathways. Moreover, modulating the cell metabolism has emerged as a new therapeutic approach to reactivate potent immune response and/or control inappropriate immune responses [5]. Some of these approaches have reached clinical development, such as arginase inhibitors for the treatment of advanced solid tumours. In HBV infection, arginase production by granulocytic myeloid-derived suppressor cells and L-arginine depletion has been associated with T cell inhibition [6], suggesting that these molecules could be of interest for the treatment of CHB patients. While specific knowledge in HBV immune-metabolism is still limited, metabolic reprogramming of exhausted T-cells during CHB has been suggested as a therapeutic strategy: HBV-specific CD8^+^ T-cells to PD-1 blockade have been shown to be improved with treatment with the acyl-CoA: cholesterol acyltransferase inhibitor (iACAT). moreover, treatment with antioxidants or polyphenolic compounds has been shown to enhance T-cell function by restoring mitochondrial function [7–9].

Aiming to further explore this concept, in the current issue of *Hepatology International*, Fu and colleagues assessed the effect of Mitochondria-Targeted Antioxidants (MTA), polyphenolic compounds, and iACATs on HBV core- and envelope (env)-specific T-cell responses *ex vivo* [10]. To address this question, the authors expanded peripheral blood mononuclear cells (PBMCs) from CHB patients of different immunological phases, namely immune-tolerant (IT), immune-active (IA), inactive carrier (IC), and HBeAg-negative hepatitis (ENEG). They found that HBV env-specific T-cells were more dysfunctional than HBV corespecific T-cells, as shown by less interferon-gamma positive HBV env-specific T-cells compared to HBV core-specific T-cells following stimulation. Furthermore, less CD8^+^ CD69^+^ CD137+ HBV env-specific T-cells were present in PBMCs than their HBV core-specific T-cells counterparts. Then, the authors showed that HBV core- and env-specific T cell responses were higher in IC and ENEG patients than IT and IA patients in their cohort (Fig. 1) [10]. The majority of patients who had no response either for HBV core-specific T cells nor for HBV env-specific T cells (double non responder) were in the IT or IA phases of the disease. These results differed from what was previously described by Le Bert and colleagues who showed progressive attrition of HBsAg-specific T cell responses in older patients with chronic hepatitis B [11]. Of note, it has been described that robust anti-core T cell responses are generally found in the presence of reduced HBsAg serum levels [12], which is the case in the IC and ENEG groups of this cohort and could account for the better immune responses observed in IC and ENEG patients. Additionally, HBeAg expression has been linked to a decrease of TLR2 expression on monocytes, leading to a skewed immune response with less of TNF-α and IFN- γ secretion, and more of anti-inflammatory cytokines, leading to suppression of HBeAg/HBcAg-specific CD8+ T-cell responses in IA and IT patients [13]. Finally, in HBeAg positive patients, higher mutations rates are observed on HBV genome, which could participate in the escape from T cell responses, even after MTA interventions [14].

Whereas the responses of HBV core-specific T cells negatively correlated with the levels of circulating HBcrAg, HBsAg, and HBV DNA, the response of HBV env-specific T cells only negatively correlated with circulating HBV DNA levels. Furthermore, using a series of *ex vivo* studies, the authors observed that whereas HBV env-specific T cells were more dysfunctional, as shown by less IFN γ -positive cells, they also responded better to metabolic interventions using MTA, iACAT, and polyphenolic compounds as shown by a greater increase of IFN γ -positive cells, compared to the HBV core-specific T cells which did not significantly respond to any of the metabolic interventions tested (Fig. 1). These results may suggest that T-cells with a more exhausted phenotype are more prone to respond to metabolic interventions, although more data would be needed for arresting conclusions.

Finally, Fu and colleagues showed that the eosinophil count and the coefficient of variation of red blood cell distribution appeared to be a predicting factor for the responsiveness of HBV env-specific T cells to metabolic interventions (i.e., MTA and polyphenolic compounds). The authors suggested that these parameters might be useful for the development of personalized therapies. However, these preliminary findings need to be validated in an independent larger cohort and their underlying mechanisms of association have to be investigated before its clinical application.

While additional studies would be required to understand the value of the findings for a therapeutic translation, this side-by-side comparison offers new insights in the ability of CHB patients to respond to metabolic interventions depending on the phase of the disease they are in. The differences in responsiveness to intervention in between the different phase of the disease suggest that this approach may need to be adapted to the patients’ viral and immune status. Moreover, when the authors compared the effect of different metabolic interventions, it appeared that MTA and polyphenolic compounds seem to have stronger efficacy in the rescue of HBV-mediated T-cell exhaustion than iACAT in their model system. Of note, Fisicaro and colleagues previously showed the efficacy of MTA to improve T-cell function and viability via correction of the excessive ROS production in these cells [2]. These promising preliminary results on T-cell function were confirmed in the present study. In a next step, it would be of interest to assess whether the absence responses to metabolic interventions were due to a lack of T-cell metabolic reprogramming or to a blockage at another level (e.g., epigenetic reprogramming).

Whereas this study provides an interesting first step for a potential future development of metabolic interventions in the treatment of CHB patients, additional studies would be required to translate this approach to the patients. The number of CHB patients recruited in this study is rather low, thus validation in an independent larger cohort would be useful to strengthen the claims. It should also be noted that HBV DNA and HBsAg levels were low and patients globally older in IC and ENEG groups compared to IT and IA patients, which could also influence the level of the T-cell response. Besides, it will be important to know whether these patients were infected at the perinatal stage or during adulthood. Additionally, it will be interesting to study the impact of HBV genotype on T-cell metabolism and on their responses to metabolic interventions. Moreover, the CHB patients recruited for this study were naïve of any treatments against CHB infection. Therefore, it would be of interest to assess the effect of the metabolic interventions analysed by the authors in CHB patients treated with standard-of-care or approaches in clinical development including longitudinal studies (e.g., early and late-stage treatment). Furthermore, since peripheral blood only partially mirrors the anti-viral T-cell responses in the liver, studies with liver-specific immune cells would be helpful to confirm the results. Overall, detailed mechanistic studies to understand the metabolic regulations occurring in T cells of CHB patients and which pathways are potentially modified by MTAs (e.g., a shift from glycolysis to mitochondrial respiration) will be needed in the future.

One of the next steps for clinical translation would be *in vivo* studies in relevant immunocompetent animal models to understand the therapeutic efficacy of the compounds not only on anti-viral immune responses but also on the viral kinetics such as viral load or cccDNA, the key determinant of viral persistence. In vivo studies would also provide information on safety, since metabolic intervention could also affect the phenotype of other immune cells, such as macrophages, immune cells particularly sensible to metabolic clues, and which functions are impaired by HBV infection [15]. Finally, as for any therapeutic approach off-target effects as single agent or combination therapy or other cells and organs including the liver in the context of HBV infection has to be taken into account. Furthermore, *in vivo* studies would enable head-to-head comparison with other immunomodulatory approaches as well as combination therapies, which are likely ultimately required for viral cure [1].

In conclusion, the study by Fu and colleagues provides another contribution in the investigation of novel targets and strategies aiming to improve the challenges of CHB treatment and ultimately cure the virus.

## Figures and Tables

**Figure 1:**
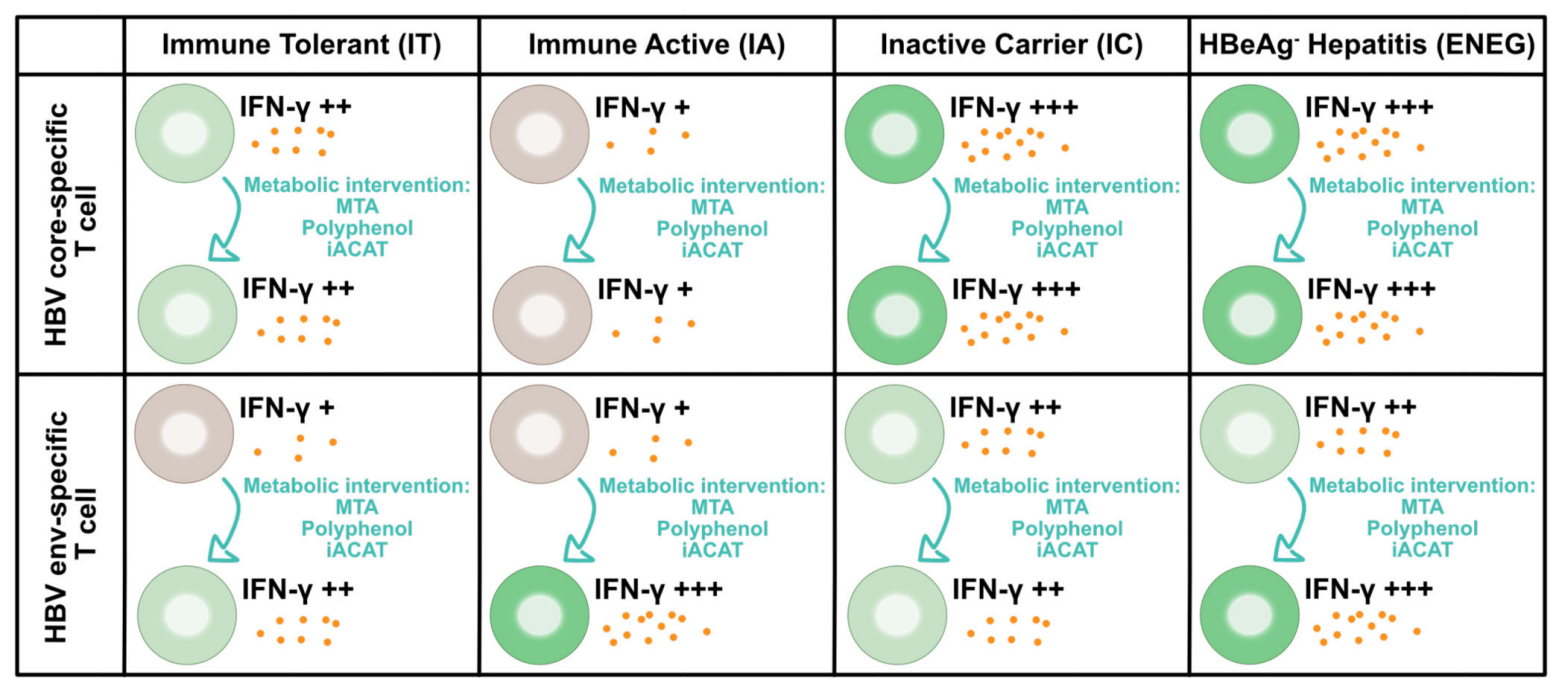
Approach and summary of key findings described by [10]. The effect of metabolic intervention on HBV core-specific and HBV env-specific T-cell exhaustion depending the phase of the disease is shown. iACAT: acyl-CoA: cholesterol acyltransferase inhibitor; IFN-γ: interferon gamma; MTA: Mitochondria-targeted antioxidants.
